# Clinical outcomes and safety of eravacycline in hematology: a multicenter, real-world study

**DOI:** 10.1128/aac.01287-25

**Published:** 2026-02-03

**Authors:** Jun Wang, Jun Zhu, Wen Wu, Rongmu Luo, Zhengyin Liu, Zhaohui Tong, Tongwen Sun, Yingchun Xu, Depei Wu

**Affiliations:** 1 Department of Hematology, The First Affiliated Hospital of Soochow University, Suzhou, China; 2 Department of Hematology, Shanghai Zhaxin Traditional Chinese & Western Medicine Hospital, Shanghai, China; 3 Department of Hematology, Ruijin Hospital Affiliated to Shanghai Jiao Tong University School of Medicine, Shanghai, China; 4 Department of Hematology, China Aerospace Science and Industry Corporation 731 Hospital, Beijing, China; 5 Department of Infectious Disease, Peking Union Medical College Hospital, Chinese Academy of Medical Sciences 34732, Beijing, China; 6 Department of Respiratory and Critical Care Medicine, Beijing Chao-Yang Hospital, Capital Medical University 74639, Beijing, China; 7 Department of Critical Care Medicine, The First Affiliated Hospital of Zhengzhou University 191599, Zhengzhou, China; 8 Department of Laboratory Medicine, State Key Laboratory of Complex Severe and Rare Diseases, Peking Union Medical College Hospital, Chinese Academy of Medical Sciences and Peking Union Medical College 12501, Beijing, China; University of Fribourg, Fribourg, Switzerland

**Keywords:** eravacycline, hematology, MDR, infectious

## Abstract

Hematology patients are highly susceptible to severe bacterial infections, particularly those caused by multidrug-resistant (MDR) gram-negative pathogens, which are associated with significant morbidity and mortality. Eravacycline, a novel fluorocycline antibiotic, demonstrates broad-spectrum activity against MDR bacteria. This real-world study aimed to evaluate the effectiveness and safety of eravacycline in Chinese hematology patients. In this multicenter, retrospective study, hematology patients receiving ≥3 days of eravacycline between September 2023 and September 2024 were included. The outcomes included clinical response rate, microbiological response rate, and safety. Of 796 patients included, most had hematological diseases (94.6%) and recent chemotherapy or radiotherapy (80.2%). The most common infection was pneumonia (57.4%), and sputum (47.2%) was the most frequent specimen type for pathogen isolation. Among 481 patients with microbiological examination results, *Klebsiella pneumoniae* (30.5%) and *Acinetobacter baumannii* (17.4%) were predominant. The mean time to defervescence was 3.2 ± 2.1 days. The overall clinical response rate was 88.8%, with response rates of 84.0% in bloodstream infections and 87.5% in pulmonary infections. Microbiological response rate at the end of treatment was 90.7%. Eravacycline exhibited high susceptibility rates across *A. baumannii* (95.8%), *K. pneumonia*e (94.3%), and *Staphylococcus aureus* (100.0%). Only 2.5% of patients reported adverse events. Subgroup analysis showed that pulmonary diseases (*P* = 0.006), sepsis (*P* = 0.003), and duration ≤7 days (*P* < 0.001) of eravacycline 1 mg/kg/12 h were significantly associated with poorer clinical response rate at the end of treatment. Eravacycline demonstrated promising effectiveness and safety in treating infections of patients from the hematology department.

## INTRODUCTION

Hematology patients are more susceptible to serious infection, of which bacterial infections—especially gram-negative—were the most clinically relevant issue, carrying increased morbidity and mortality (1, 2). Despite preferred antibiotics recommended in the clinical practice, the emergence of multidrug-resistant (MDR) gram-negative bacilli, such as *Escherichia coli* and *Klebsiella pneumoniae*, has complicated the optimal treatment option and posed a significant challenge to clinicians and public health (1, 3, 4). In the context of the urgent global crisis of antimicrobial-resistant infections, new antibiotic therapy with a wider range of activity is needed.

Eravacycline is the first fully synthetic fluorocycline and exerts broad-spectrum activity against anaerobes, gram-negative bacteria, and gram-positive bacteria, such as extended-spectrum β-lactamase (ESBL)-producing Enterobacterales, carbapenem-resistant Enterobacterales (CRE), methicillin-resistant *Staphylococcus aureus* (MRSA), and vancomycin-resistant enterococci (5, 6). Based on the non-inferiority from the IGNITE 1 and IGNITE 4 trials, eravacycline has received approval for the treatment of complicated intra-abdominal infections (cIAIs) (7, 8). Although several retrospective studies provided valuable evidence for clinical practice of eravacycline outside the prospective trial settings, real-world evidence remains limited, with the small sample size and the absence of Asian experience and data on clinically relevant infections in hematology (9–12). In the present study, we retrospectively evaluated the effectiveness and safety of eravacycline in hematology patients with multidrug-resistant infections across China.

## MATERIALS AND METHODS

### Study design and sample

This retrospective, multicenter, real-world study included patients from the department of hematology between September 2023 and September 2024 at 71 centers across China. Eligible patients received eravacycline for at least 3 days. Patients who participated concurrently in clinical trials or received eravacycline prophylactically only were excluded from this study.

### Data collection and outcomes

We retrospectively collected the patient data from electronic medical records, including baseline characteristics (underlying diseases and infection sites), microbiological data (pathogen identification and antimicrobial susceptibility results), details of treatment regimens (including dosage, treatment duration, and combination therapies), infection-related symptoms, signs, auxiliary examinations, and any adverse events (AEs). Patients’ records were manually reviewed to confirm drug administration and documentation of adverse events. Data were then pooled into a single secure database.

The outcomes included clinical response rate (defined as complete resolution of infection-related symptoms, signs, and ancillary tests, with confirmed or presumed pathogen eradication, or as significant improvement in these parameters with at least one not fully normalized), microbiological response rate (defined as pathogen eradication, presumed eradication, or microbial substitution [replacement by non-primitive pathogens]), and safety. Normal body temperature was defined as a temperature of <37.3°C maintained for at least 24 h, based on both Chinese and international infectious disease criteria. Mixed-microbiological infection was defined as the isolation of two or more different pathogenic microorganisms from the same infection site or specimen.

For minimum inhibitory concentrations (MICs), all cultures, bacterial identifications, and antibiotic susceptibilities were conducted according to local procedures at each center. Clinical Laboratory Standards Institute (CLSI)/European Committee on Antimicrobial Susceptibility Testing (EUCAST)/Food and Drug Administration (FDA)/China Antimicrobial Susceptibility Testing (ChinaCAST) breakpoints were used to interpret MIC results, where applicable. AEs were monitored and recorded by the investigators, and the severity was graded according to the National Cancer Institute Common Terminology Criteria for Adverse Events (NCI-CTCAE) version 5.0.

### Statistical analysis

Descriptive statistics were employed to evaluate baseline characteristics. Frequencies and percentages were used to report categorical variables, while continuous data were described using median and interquartile range (IQR) or mean and standard deviation, depending on the normality of the distribution. Statistical comparisons of clinical response rates according to baseline characteristics were performed using the chi-squared test. All statistical tests were two-sided, with significance set at *P* values of <0.05. IBM SPSS Statistics version 29 (IBM Corp., Armonk, NY) was used to carry out the analysis.

## RESULTS

### Baseline characteristics

From September 2023 to September 2024, 796 patients receiving ≥3 days of eravacycline in the department of hematology were included. Baseline characteristics are summarized in Table 1. The median age was 49.0 (range, 18–89) years, and the majority of the patients were male (58.9%). Hematological diseases (94.6%, mainly including acute myeloid leukemia and acute lymphoblastic leukemia), pulmonary diseases (57.2%), and cardiovascular diseases (14.3%, mainly including hypertension and cardiac insufficiency) were the most common comorbidities. Almost all the patients had high risk factors associated with the immunosuppression, the most common being chemotherapy/radiotherapy within 1 month (80.2%). Over half of the patients were diagnosed with pneumonia (57.4%). Half of the patients (50.5%) underwent eravacycline monotherapy, and the rest (49.5%) underwent eravacycline-based combination therapies. The median scores of sequential organ failure assessment (SOFA) were 2.5 (IQR, 2.0–4.0). Almost all (97.7%) patients received the standard dose of 1 mg/kg/12 h. The average administration period of eracycline was 8.9 (range, 3–52) days.

**TABLE 1:** Baseline characteristics^*d*^

Characteristic	Patients, *n* (%) *N* = 796
Age (years), median (range)	49.0 (18.0–89.0)
Male	469 (58.9)
Comorbidities	
Hematological diseases	753 (94.6)
Acute myeloid leukemia	345 (43.3)
Acute lymphoblastic leukemia	79 (9.9)
Others	372 (46.7)
Pulmonary diseases	455 (57.2)
Cardiovascular diseases	114 (14.3)
Hypertension	67 (8.4)
Cardiac insufficiency	13 (1.6)
Others	34 (4.3)
Neutropenia	594 (74.6)
Sepsis	101 (12.7)
Diabetes	80 (10.1)
Kidney disease	59 (7.4)
Nervous system disease	21 (2.6)
Rheumatic immune diseases	20 (2.5)
Other underlying diseases and comorbidities	110 (13.8)
High risk factor associated with immunosuppression	
Chemotherapy/radiotherapy within 1 month	638 (80.2)
Long-term use of corticosteroids/immunosuppressants	460 (57.8)
Transplantation	260 (32.7)
AIDS	5 (0.6)
Splenectomy	6 (0.8)
Infection sites^*a*^	
Pneumonia	457 (57.4)
Abdominal infection	48 (6.0)
Bloodstream infections	58 (7.3)
Pneumonia + bloodstream infections	71 (8.9)
Pneumonia + abdominal infection	30 (3.8)
Abdominal infection + bloodstream infection	5 (0.6)
Pneumonia + abdominal infection + bloodstream infection	8 (1.0)
Others	119 (14.9)
Laboratory tests, median (IQR)	
White blood cell count (×10^9^/L)	2.0 (0.4–5.8)
Outlier^*b*^	640 (80.4)
Neutrophil count (×10^9^/L)	1.0 (0.1–3.5)
Outlier^*b*^	578 (72.6)
Neutrophil ratio (%)	53.7 (20.0–79.6)
Outlier^*b*^	544 (68.3)
C-reactive protein (mg/L)	54.7 (19.5–111.6)
Outlier^*b*^	676 (84.9)
Procalcitonin (ng/mL)	0.7 (0.2–2.6)
Outlier^*b*^	787 (98.9)
SOFA score, median (IQR)	2.5 (2.0–4.0)
Eravacycline-based regimen	
Monotherapy^*c*^	402 (50.5)
Combination^*c*^	394 (49.5)
Eravacycline + polymyxins	70 (8.8)
Eravacycline + carbapenems	15 (1.9)
Eravacycline + aminoglycosides	8 (1.0)
Eravacycline + ceftazidime-avibactam	2 (0.3)
Other	65 (8.2)
Unknown	234 (29.4)
Dosage of eravacycline	
1 mg/kg q12h	778 (97.7)
≤7 days	370 (46.5)
7–14 days	287 (36.1)
>14 days	121 (15.2)
50 mg q12h	15 (1.9)
≤7 days	8 (1.0)
7–14 days	6 (0.8)
>14 days	1 (0.1)
Other	3 (0.4)

^*a*^ Other sites include oral mucositis, skin and soft tissue infections, respiratory tract infections, and other less common infection sites.

^*b*^ Outliers represent the number of patients with values outside the normal range. Normal ranges were defined as follows: white blood cell count = 3.5–9.5 × 10⁹/L, neutrophil count = 1.8–6.3 × 10⁹/L, neutrophil ratio = 40%–75%, C-reactive protein ≤10 mg/L, procalcitonin <0.05 ng/mL.

^*c*^ Monotherapy was defined as receipt of only eravacycline for eravacycline-targeted pathogens, and combined therapy was defined as the concurrent use of eravacycline with one or more additional antibiotics.

^*d*^ Data represent the number of patients with outlier values. AIDS, acquired immune deficiency syndrome; IQR, interquartile range; SOFA, sequential organ failure assessment.

### Microbiological characteristics

**Fig 1:**
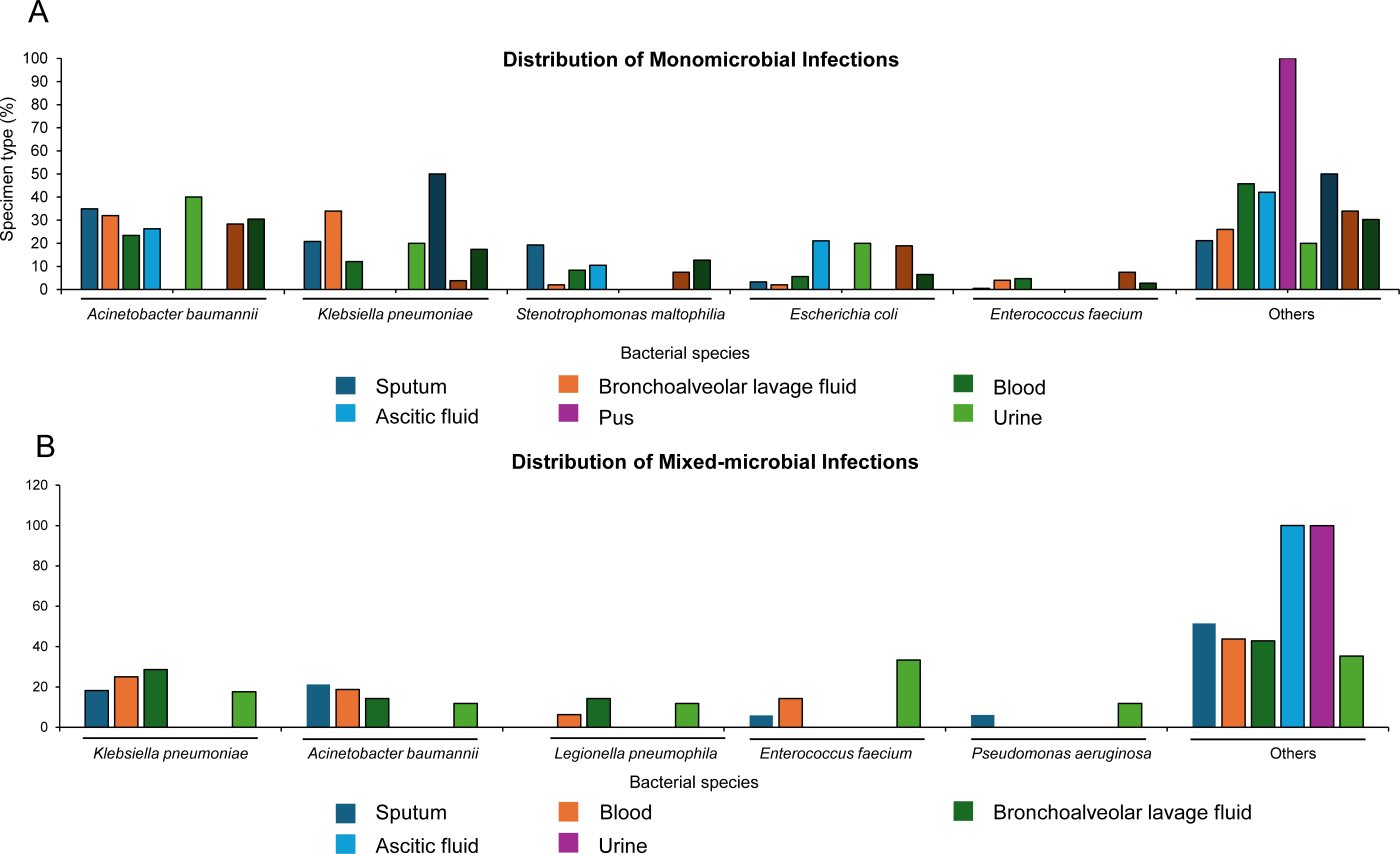
Distribution of monomicrobial infections (**A**) and mixed-microbial infections (**B**).

Among the 481 patients with microbiological examination results, 446 (92.7%) were infected by a single pathogen, and 35 (7.3%) were infected by mixed pathogens. Among single-pathogen infections, *Acinetobacter baumannii* was the most common (30.5%), followed by *K. pneumoniae* (17.4%), *Stenotrophomonas maltophilia* (12.7%), *E. coli* (6.5%), and *Enterococcus faecium* (2.7%). The predominant source of *A. baumannii* was sputum (*n* = 74), followed by blood (*n* = 25) and bronchoalveolar lavage fluid (*n* = 16). *K. pneumoniae* spp. were mainly derived from sputum (*n* = 44) and bronchoalveolar lavage fluid (*n* = 17, Fig. 1A and Table S1). Of 76 mixed-pathogen infections, the most common pathogens were *K. pneumoniae* (19.7%), *A. baumannii* (17.1%), *E. faecium* (11.1%), *Legionella pneumophila* (5.3%), and *Pseudomonas aeruginosa* (5.3%), with the predominant source of sputum (*n* = 33, Fig. 1B and Table S2).

### Clinical and microbiological effectiveness

A total of 732 patients had available data on defervescence. The mean time to return to normal body temperature was 3.2 ± 2.1 days, with no significant difference observed in patients receiving eravacycline combination. Laboratory test indicators, including C-reactive protein, procalcitonin, and SOFA score, were decreased both at 3 days and at the end of treatment (Fig. S1).

**Fig 2:**
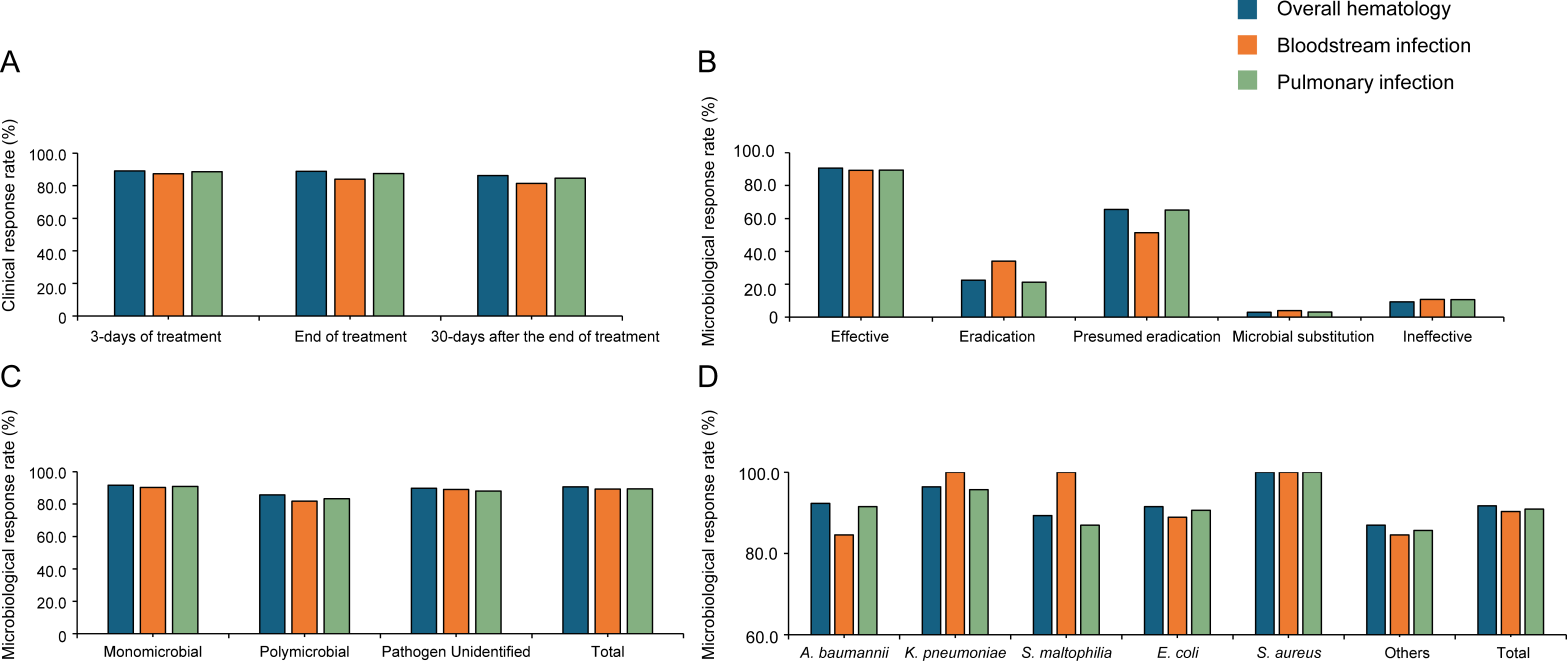
Clinical and microbiological response rates in patients with different infection sites. (**A**) Clinical response rates at different time points. (**B**) Microbiological response rates in patients with different infection sites. (**C**) Microbiological response rates in monomicrobial and polymicrobial pathogen infections. (**D**) Microbiological response rates in different pathogen species. Abbreviations: *A. baumannii*, *Acinetobacter baumannii*; *K. pneumoniae*, *Klebsiella pneumoniae*; *S. maltophilia*, *Stenotrophomonas maltophilia*; *E. coli*, *Escherichia coli*; *S. aureus*, *Staphylococcus aureus*.

The clinical response rates at 3 days, the end, and 30 days after the end of eravacycline treatment were 89.1%, 88.8%, and 86.2%, respectively. At 3 days, 79 patients (9.9%) showed no clinical response, and 8 (1.0%) had died. At the end of treatment, 59 (7.4%) were non-responsive and 30 (3.8%) had died. Within 30 days after the end of eravacycline treatment, 33 (4.1%) remained non-responsive and 75 (9.4%) deaths occurred. Patients with bloodstream infections achieved clinical response rates of 87.3% at 3 days, 84.0% at the end of treatment, and 81.4% at 30 days after the end of treatment. In patients with pulmonary infections, the clinical response rates were 88.6%, 87.5%, and 84.6% at the corresponding time points, respectively (Fig. 2A). At the end of treatment, the overall microbiological response rate was 90.7%, with a pathogen eradication rate of 22.4% and a presumed eradication rate of 65.5% (Fig. 2B). When stratified by infection site, patients achieved microbiological response rates of 91.7% in monomicrobial infections and 85.7% in polymicrobial infections (Fig. 2C). Eravacycline demonstrated both clinical and microbiological response rates of *A. baumannii* and *K. pneumoniae* (Fig. 2D; Fig. S2).

Subgroup analysis showed that pulmonary diseases (*P* = 0.005), other underlying diseases and comorbidities (*P* = 0.009), monotherapy (*P* = 0.007), combination (*P* = 0.007), eravacycline treatment at a dosage of 1 mg/kg/12 h for ≤7 days (*P* < 0.001) and 7–14 days (*P* < 0.001) were significantly associated with clinical response rate after 3 days, and pulmonary diseases (*P* = 0.006), sepsis (*P* = 0.003), duration ≤7 days (*P* < 0.001) and between 7–14 days (*P* < 0.001) of 1 mg/kg/12 h eravacycline treatment showed significant associations for clinical response rate at the end of treatment (Table 2). At the eravacycline dosage of 1 mg/kg/12 h, clinical response rate was significantly higher with a treatment duration of 7–14 days than with <7 days, both at 3 days and at the end of treatment.

**TABLE 2:** Subgroup analysis^*b*^

Factor	*n* (%)	After 3 days		End of treatment	
		Clinical response rate (%)	*P* value	Clinical response rate (%)	*P* value
Sex			0.527	88.8	0.432
Male	469 (58.9)	88.5		89.6	
Female	327 (41.1)	89.9		87.8	
Comorbidities					
Hematological diseases	753 (94.6)	89.2	0.513	88.8	0.924
Pulmonary diseases	455 (57.2)	86.4	0.005	86.2	0.006
Sepsis	101 (12.7)	86.1	0.312	80.2	0.003
Other underlying diseases and comorbidities	110 (13.8)	81.8	0.009	88.2	0.819
Infection sites					
Pneumonia	457 (57.4)	88.4	0.483	88.8	0.982
Abdominal infection	48 (6.0)	87.5	0.719	91.7	0.518
Bloodstream infections	58 (7.3)	87.9	0.773	91.4	0.52
Laboratory tests^*a*^					
White blood cell count	640 (80.4)	88.6	0.383	88.9	0.874
Neutrophil count	578 (72.6)	88.8	0.642	89.1	0.682
Neutrophil ratio	544 (68.3)	87.9	0.11	87.9	0.211
C-reactive protein	677 (85.1)	88.9	0.698	88.2	0.156
Procalcitonin	787 (98.9)	89.1	0.986	88.7	0.284
SOFA score	426 (53.5)	86.9	0.032	88.5	0.757
<Median	213 (50.0)	89.2	0.013	91.1	0.116
≥Median	213 (50.0)	84.5	0.013	85.9	0.116
Eravacycline-based regimen					
Monotherapy	402 (50.5)	92.0	0.007	90.8	0.074
Combination	394 (49.5)	86.0	0.007	86.8	0.074
Dosage of eravacycline					
1 mg/kg q12h	778 (97.7)	88.8	0.133	88.8	0.992
≤7 days	370 (46.5)	83.0	<0.001	82.2	< 0.001
7–14 days	287 (36.1)	96.9	<0.001	95.5	< 0.001
>14 days	121 (15.2)	87.6	0.574	93.4	0.083
50 mg q12h	15 (1.9)	100.0	0.171	86.7	0.789
≤7 days	8 (1.0)	100.0	0.319	75.0	0.213
7–14 days	6 (0.8)	100.0	0.389	100.0	0.383
>14 days	1 (0.1)	100.0	0.726	100.0	0.723

^*a*^ Data represent the number of patients with outlier values.

^*b*^ SOFA, sequential organ failure assessment.

### Antimicrobial susceptibility test results

**Fig 3:**
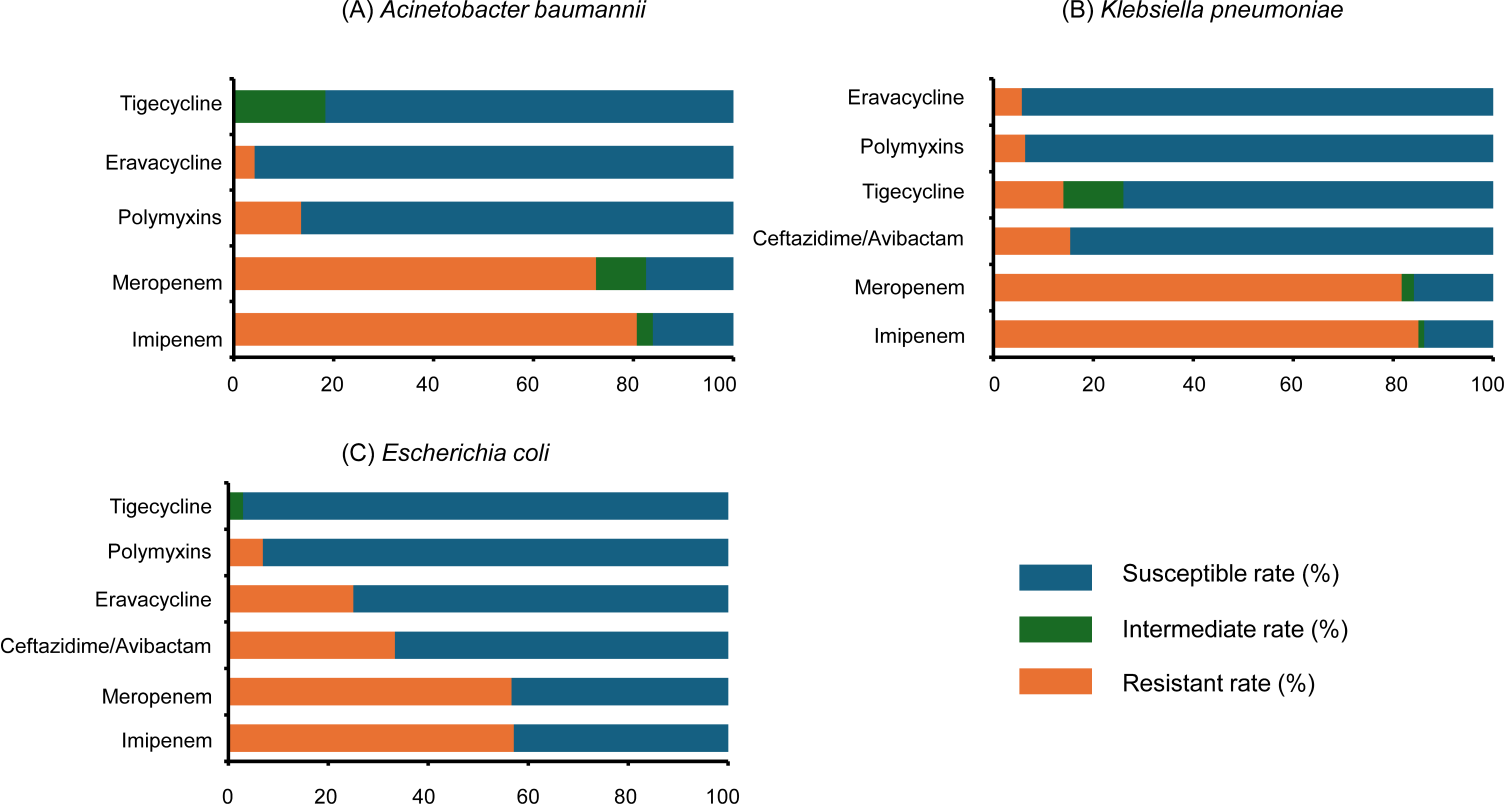
Susceptibility rates of pathogens to antimicrobial agents. (**A**) *Acinetobacter baumannii.* (**B**) *Klebsiella pneumoniae.* (**C**) *Escherichia coli.*

The susceptibility rates of *A. baumannii* to eravacycline, tigecycline, and polymyxins were 94.3%, 81.7%, and 93.7%. The resistant rates of *A. baumannii* to imipenem and meropenem were 80.6% and 72.5% (Fig. 3A). *K. pneumoniae* showed susceptibility to eravacycline and tigecycline of 94.3% and 74.0%, with similar resistance to both imipenem (85.1%) and meropenem (81.7%; Fig. 3B). Eravacycline and tigecycline showed strong activity against *E. coli*, with susceptibility rates of 75.0% and 97.1%, respectively. *E. coli* displayed resistance to carbapenems, with 57.1% resistant to imipenem and 56.7% to meropenem (Fig. 3C). *S. aureus* demonstrated full susceptibility to both eravacycline and tigecycline (Fig. 3). *S. maltophilia* susceptibility to eravacycline could not be formally interpreted due to limited data and absence of established criteria. However, its MIC values were low overall, with a median MIC of 0.13 μg/mL, MIC_50_ of 0.5 μg/mL, MIC_90_ of 0.75 μg/mL, and inhibition zone diameters ranging from 6 to 30 mm.

### Safety

Detailed AEs are summarized in Table 3. Among the 796 patients included in this study, 20 patients (2.5%) reported AEs, of whom 13 (65%) had received an eravacycline-based combination. The most frequent AEs were nausea (1.5%), vomiting (1.5%), increased hepatic enzyme (0.9%), infusion reaction (0.9%), and rash (0.6%). After eravacycline treatment, 69 (8.7%) patients underwent rescue therapy with other antibiotics. Thirteen patients (1.6%) experienced organ failure. Seventy-seven patients (9.7%) were admitted to the intensive care unit due to infection or infection-related complications, with a median length of stay of 7 (range, 1–134) days. A total of 84 patients (10.6%) died, including 30 (3.8%) due to infections and 54 (6.8%) due to non-infectious causes.

**TABLE 3:** Adverse events of any grade

Adverse event	Any grade, *n* (%)
Any adverse event	20 (2.5)
Nausea	12 (1.5)
Vomiting	12 (1.5)
Hepatic enzyme increased	7 (0.9)
Infusion reaction	5 (0.6)
Rash	2 (0.3)
Hypocalcemia	1 (0.1)
Dizziness	1 (0.1)
Amylase increased	1 (0.1)
Lipase increased	1 (0.1)

## DISCUSSION

This study provides the first antibacterial profile evidence supporting the clinical and microbiological effectiveness and safety of eravacycline, particularly in hematology patients with infections. Our study has demonstrated that eravacycline showed clinically promising effectiveness and safety, with the clinical response rate and microbiological response rate at 88.8% and 90.7%, respectively. Eravacycline showed high susceptibility rates against MDR pathogens, such as *A. baumannii* and *K. pneumoniae*. Infrequent AEs and no new safety signals were observed, indicating that eravacycline was administered safely in hematology patients. To our knowledge, it represents the largest real-world observational investigation of eravacycline conducted in China to date. It is expected that the accumulation of clinical experience and data from China will contribute to the global evidence on eravacycline for clinical application and informing antimicrobial stewardship worldwide, ultimately benefiting patients.

Among the 796 patients from the hematology department, eravacycline, administered either as monotherapy or in combination, demonstrated an overall clinical response rate of 88.8%, which was comparable to the reported effectiveness of conventional carbapenems (7, 8, 13, 14). To further evaluate its pathogen-specific effectiveness beyond general clinical outcomes, we analyzed the microbiological response rates. Microbiological effectiveness analysis showed overall response rates of 90.7%, with 91.7% for monomicrobial infections and 85.7% for polymicrobial infections, indicating that eravacycline is effective both as monotherapy and in combination regimens against a broad range of pathogens. For patients in critical condition, combination regimens are recommended. Given that patients in this study presented with multiple concurrent infections and resistance to various gram-negative pathogens, the observed clinical effectiveness of eravacycline is especially meaningful—particularly in the context of patients with multiple underlying comorbidities. The majority of patients (83.9%) were eventually discharged with clinical improvement, with a small proportion of deaths due to infections (30/796, 3.8%). Additionally, the IGNITE1 and IGNITE4 trials were limited to a specific infection site (i.e., cIAI) rather than other sites with more common *A. baumannii* infections, such as pulmonary, blood, and urine (9). In comparison, our study enriches the limited real-world data in Asian populations with clinical response rates of 84.0% and 87.5% in patients with bloodstream and pulmonary infections, respectively, supporting the potential of eravacycline as a treatment option for challenging infections such as CRE, ESBL, MRSA, and *S. maltophilia*.

Early defervescence is frequently regarded as a surrogate marker of treatment effectiveness (15). The mean time to return to normal body temperature was 3.2 ± 2.1 days in our study, indicating a relatively rapid response to eravacycline. The observed result supports the clinical utility of eravacycline in hematology patients who are often immunocompromised and at elevated risk of infection-related complications (15–17). Subgroup analysis further revealed that the duration of eravacycline therapy had a significant impact on clinical outcomes. Specifically, treatment durations of ≤7 days at the standard dose of 1 mg/kg were associated with lower clinical response rates (*P* < 0.001), while patients receiving eravacycline for 7–14 days exhibited significantly improved clinical response rates (*P* < 0.001). These results prompt us to consider optimizing treatment duration based on individual patient factors such as infection severity, pathogen resistance profiles, and immunologic status. The findings regarding antibiotic duration should be interpreted with caution due to the retrospective design of the study. Nevertheless, ensuring adequate dosage and a full course of treatment remains essential to achieving optimal therapeutic outcomes.

Clinical guidelines have reported the limited susceptibility of imipenem and meropenem against gram-negative pathogens, particularly *K. pneumoniae* and *A. baumannii* (18, 19). Consistently, our study also identified high resistance rates to both carbapenems among clinical isolates. Nevertheless, we also observed favorable clinical effectiveness of eravacycline despite the high prevalence of carbapenem resistance. In contrast, eravacycline demonstrated higher *in vitro* susceptibility against *K. pneumoniae* and *A. baumannii*, surpassing even tigecycline, another commonly used tetracycline antibiotic. Structurally, eravacycline is a synthetic fluorocycline antibacterial agent closely related to tigecycline, with two key modifications on the D-ring of its tetracycline core: a fluorine atom replaces the dimethylamine moiety at C-7, and a pyrrolidinoacetamido group replaces the 2-tertiary-butyl glycylamido at C-9. These structural enhancements confer two- to fourfold greater potency against most aerobic gram-negative pathogens than tigecycline (20). This structural advantage may help explain the higher susceptibility rates and the promising clinical effectiveness observed in our study. In addition, our study observed that *E. coli* showed higher susceptibility to tigecycline compared to eravacycline (97.1% vs 75.0%). This finding may be attributed to the use of the FDA breakpoints (2, 4, and 8 mg/L) for tigecycline, which could potentially overestimate its susceptibility, as the EUCAST has lowered the clinical susceptibility breakpoint of tigecycline to ≤0.5 mg/L. Thus, susceptibility data should be interpreted with caution, considering the differences in breakpoint standards and their clinical implications.

In terms of safety, 20 out of 796 patients (2.5%) reported any AEs. The most frequently observed AEs included gastrointestinal disorders (nausea [1.5% vs 4.8%−8.1%] and vomiting [1.5% vs 3.6%]), and hepatic enzyme increased (0.9%), lower than the known safety profile reported in the IGNITE 1 and 4 trials, with no new safety signals (7, 8). Compared with previous clinical trials and real-world analysis of other antibiotic and tetracyclines, the incidence of AEs in this study, such as nausea and vomiting, was far lower, even in patients receiving combination regimens, suggesting the favorable tolerability of eravacycline in real-world settings (5, 7, 8, 21–25).

Certain limitations of our study should be acknowledged. First, the selection biases were inevitable due to the non-randomized and retrospective nature of the study. Second, although centralized microbiological testing was not performed, standardized methods for microbiological testing were adopted across all centers. Despite its imperfections, these real-world data may provide complementary references for clinicians in hematology by reflecting the performance of eravacycline in routine clinical practice. Future prospective multicenter studies are warranted to further validate these findings and define the optimal use of eravacycline within antimicrobial stewardship frameworks.

### Conclusion

In conclusion, eravacycline demonstrated good effectiveness and safety in treating infections of patients in the hematology department. Given the limited clinical experience with eravacycline in Chinese populations, our study may provide valuable real-world evidence to guide clinical decision-making.

## Data Availability

The data sets generated and analyzed during the current study are available from the corresponding author on reasonable request.

## SUPPLEMENTAL MATERIAL

Supplemental material aac.01287-25-s0001.docx Tables S1 and S2; Fig. S1 and S2.

ASM does not own the copyrights to Supplemental Material that may be linked to, or accessed through, an article. The authors have granted ASM a non-exclusive, world-wide license to publish the Supplemental Material files. Please contact the corresponding author directly for reuse.
