# Supplementary material for: Clinical outcomes and safety of eravacycline in hematology: a multicenter, real-world study
Source: Antimicrob Agents Chemother. 2026 Feb 3;70(3):e01287-25. doi: 10.1128/aac.01287-25 (PMC12959114; doi:10.1128/aac.01287-25)
Supplement: Supplemental material — Tables S1 and S2; Fig. S1 and S2. [file aac.01287-25-s0001.docx]

**Supplementary Materials**

**Supplementary table S1．Sources of single pathogen.**

| Species of single bacteria | Type of positive culture^*^, n (%) | | | | | | | | |
| --- | --- | --- | --- | --- | --- | --- | --- | --- | --- |
|  | Sputum (n=212) | Bronchoalveolar lavage fluid (n=50) | Blood (n=107) | Ascitic fluid (n=19) | Pus (n=1) | Urine (n=5) | Pleural effusion (n=2) | Others (n=53) | Total (n=449) |
| *A. baumannii* | 74 (34.9) | 16 (32.0) | 25 (23.4) | 5 (26.3) | 0 | 2 (40.0) | 0 | 15 (28.3) | 137 (30.5) |
| *K. pneumoniae* | 44 (20.8) | 17 (34.0) | 13 (12.1) | 0 | 0 | 1 (20.0) | 1 (50.0) | 2 (3.8) | 78 (17.4) |
| *S. maltophilia* | 41 (19.3) | 1 (2.0) | 9 (8.4) | 2 (10.5) | 0 | 0 | 0 | 4 (7.5) | 57 (12.7) |
| *E. coli* | 7 (3.3) | 1 (2.0) | 6 (5.6) | 4 (21.1) | 0 | 1 (20.0) | 0 | 10 (18.9) | 29 (6.5) |
| *E. faecium* | 1 (0.5) | 2 (4.0) | 5 (4.7) | 0 | 0 | 0 | 0 | 4 (7.5) | 12 (2.7) |
| Others | 45 (21.2) | 13 (26.0) | 49 (45.8) | 8 (42.1) | 1 (100.0) | 1 (20.0) | 1 (50.0) | 18 (34.0) | 136 (30.3) |
| Total | 212 (47.2) | 50 (11.1) | 107 (23.8) | 19 (4.2) | 1 (0.2) | 2 (1.1) | 2 (0.4) | 53 (11.8) | 449 (100.0) |

**^*^**Data were calculated based on the number of patients with corresponding specimens.

**Abbreviation:** *A. baumannii*, *Acinetobacter baumannii*; *K. pneumoniae*, *Klebsiella pneumoniae*; *S. maltophilia*, *Stenotrophomonas maltophilia*; *E. coli*, *Escherichia coli*; *E. faecium*, *Enterococcus faecium*.

**Supplementary table S2．Sources of mixed pathogen.**

| Species of mixed bacteria | Type of positive culture^*^, n (%) | | | | | | |
| --- | --- | --- | --- | --- | --- | --- | --- |
|  | Sputum (n=33) | Blood (n=16) | Bronchoalveolar lavage fluid (n=7) | Ascitic fluid (n=2) | Urine (n=1) | Others (n=17) | Total (n=76) |
| *K. pneumoniae* | 6 (18.2) | 4 (25.0) | 2 (28.6) | 0 | 0 | 3 (17.6) | 15 (19.7) |
| *A. baumannii* | 7 (21.2) | 3 (18.8) | 1 (14.3) | 0 | 0 | 2 (11.8) | 13 (17.1) |
| *L. pneumophila* | 0 | 1 (6.3) | 1 (14.3) | 0 | 0 | 2 (11.8) | 4 (5.3) |
| *E. faecium* | 1 (5.9) | 1 (14.3) | 0 | 0 | 0 | 2 (33.3) | 4 (11.1) |
| *P. aeruginosa* | 2 (6.1) | 0 | 0 | 0 | 0 | 2 (11.8) | 4 (5.3) |
| Others | 17 (51.5) | 7 (43.8) | 3 (42.9) | 1 (100.0) | 1 (100.0) | 6 (35.3) | 36 (47.4) |
| Total | 33 (43.4) | 16 (21.1) | 7 (9.2) | 1 (1.3) | 1 (1.3) | 17 (22.4) | 76 (100.0) |

**^*^**Data were calculated based on the number of patients with corresponding specimens.

**Abbreviation:** *K. pneumoniae*, *Klebsiella pneumoniae*; *A. baumannii*, *Acinetobacter baumannii*; *L. pneumophila*, *Legionella pneumophila*; *E. faecium*, *Enterococcus faecium; P. aeruginosa, Pseudomonas aeruginosa.*


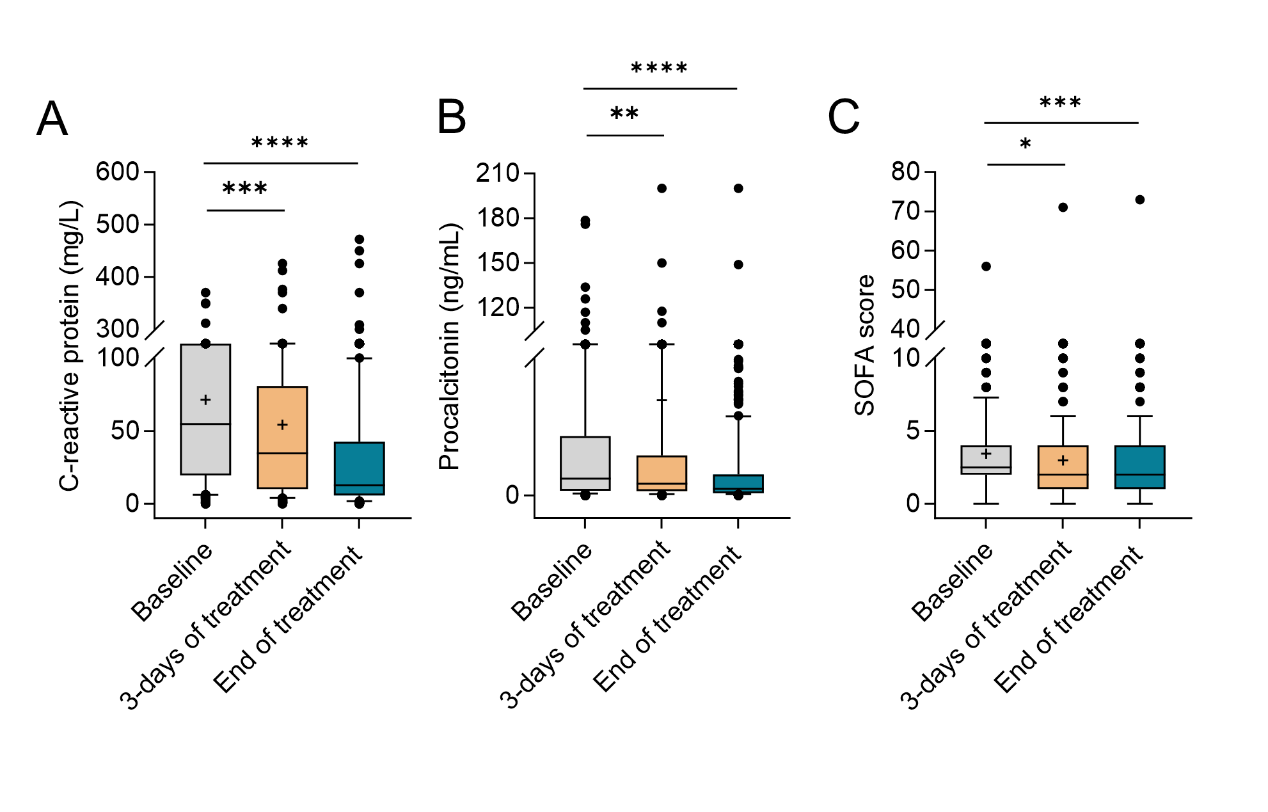


**Supplementary figure S1.** Changes of laboratory test indicators at 3-days and at the end of treatment. (A) C-reactive protein. (B) Procalcitonin. (C) SOFA score.

**Abbreviations:** SOFA, sequential organ failure assessment.


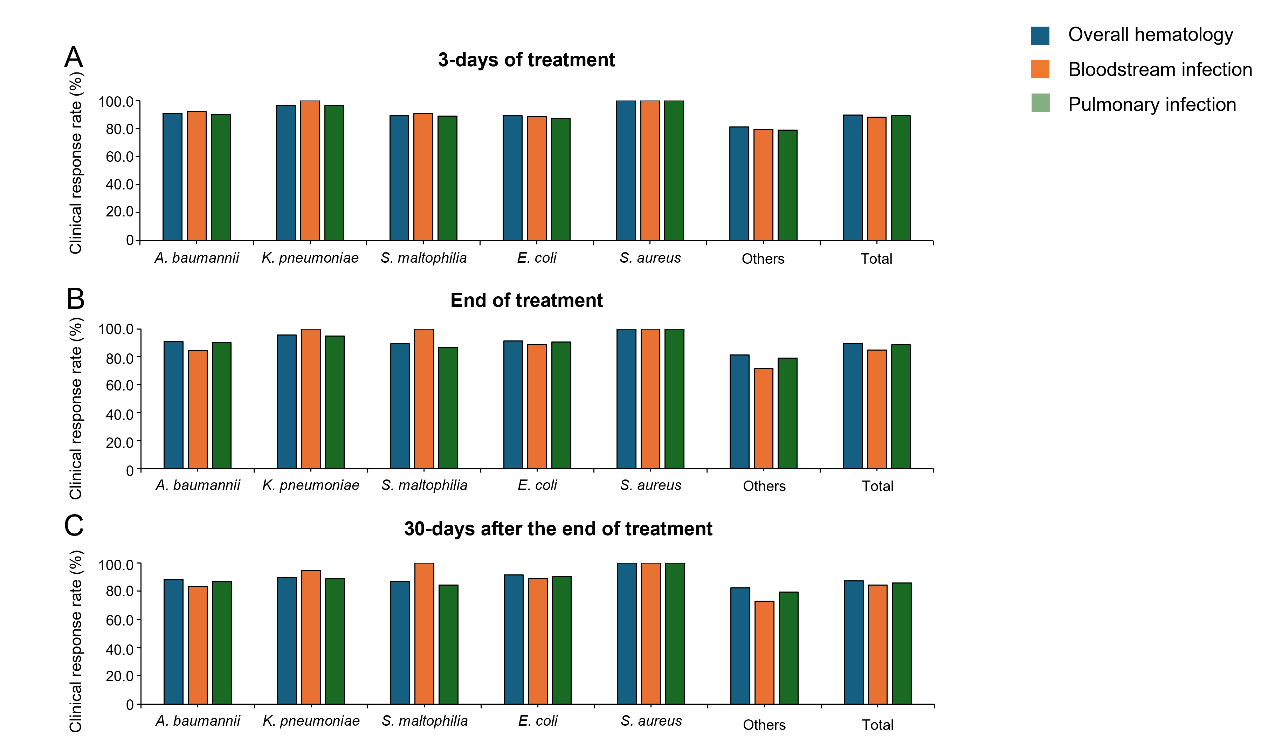


**Supplementary figure S2. Clinical response rates in different pathogen species at different timepoints.** (A) 3-days of treatment. (B) End of treatment. (C) 30-days after the end of treatment.

**Abbreviations:** *A. baumannii, Acinetobacter baumannii; K. pneumoniae, Klebsiella pneumoniae; S. maltophilia, Stenotrophomonas maltophilia; E. coli, Escherichia coli; S. aureus, Staphylococcus aureus.*
